# An Improved Inertial Frame Alignment Algorithm Based on Horizontal Alignment Information for Marine SINS

**DOI:** 10.3390/s151025520

**Published:** 2015-10-05

**Authors:** Yanting Che, Qiuying Wang, Wei Gao, Fei Yu

**Affiliations:** 1 College of Automation, Harbin Engineering University, Harbin, China; E-Mail: yufei@hrbeu.edu.cn; 2 College of Information and Communication Engineering, Harbin Engineering University, Harbin, China; E-Mail: wqy869087@163.com

**Keywords:** SINS, alignment, dimension reduction GHF, inertial coordinate frame

## Abstract

In this paper, an improved inertial frame alignment algorithm for a marine SINS under mooring conditions is proposed, which significantly improves accuracy. Since the horizontal alignment is easy to complete, and a characteristic of gravity is that its component in the horizontal plane is zero, we use a clever method to improve the conventional inertial alignment algorithm. Firstly, a large misalignment angle model and a dimensionality reduction Gauss-Hermite filter are employed to establish the fine horizontal reference frame. Based on this, the projection of the gravity in the body inertial coordinate frame can be calculated easily. Then, the initial alignment algorithm is accomplished through an inertial frame alignment algorithm. The simulation and experiment results show that the improved initial alignment algorithm performs better than the conventional inertial alignment algorithm, and meets the accuracy requirements of a medium-accuracy marine SINS.

## 1. Introduction

A strapdown inertial navigation system (SINS) is a dead-reckoning navigation system, and the initial alignment is an essential procedure for a SINS, since it directly affects the precision of navigation parameters (position, velocity, and attitude) [1,2]. The main purpose of the initial alignment is to determine the initial strapdown attitude matrix between the body frame and navigation frame, and its accuracy is especially important for a marine SINS, which usually has to work for a long time [1,2,3,4].

Generally, the alignment process can be divided into two phases, the coarse-alignment phase and the fine-alignment phase [4,5,6,7,8]. The coarse-alignment phase is required to estimate the ship’s heading within a few degrees and pitch/roll within a few tenths of a degree in order to allow the fine-alignment filter to operate within its linear region [5,6]. The typical coarse-alignment method is analytic coarse-alignment. However, it is unable to handle the in-motion alignment problem [3,4,5,7]. In order to overcome the difficulties when a marine SINS is under mooring conditions, many methods have been developed and analyzed. Reference [3] proposed an improved alignment based on gravity in an inertial frame, and velocity is used in the calculation to reduce the influence of disturbance acceleration. In mooring conditions, due to the presence of the disturbed acceleration and angular velocities, accurate gravity and Earth rate are difficult to obtain directly, which finally leads to the low precision of the coarse alignment. Since the signal-to-noise ratios of gyros’ and accelerometers’ output are poor and the frequency bands of disturbed signals are wide, it is unable to separate the pure, useful signals from the interference signals measured by gyros and accelerometers [6,7,9]. In order to remove the high frequency noise, reference [7] used an IIR digital low-pass filter to process the gyro and accelerometer measurements. Although there have been various methods presented so as to obtain the purer Earth rate and gravity signals, the precision is still not high enough.

While in the fine-alignment phase, usually the standard Kalman filter or the compass loop method can be implemented based on the coarse-alignment result, and under the assumption of a small misalignment angle linear error model [1,2]. However, with the increase of SINS application technology and the development of nonlinear filtering estimation technology, its error models are no longer confined to linear models, and new error models are constantly emerging. Therefore, nonlinear filters are used for alignment [10,11,12,13,14,15,16]. However, no matter what method is employed, the heading misalignment angle will usually converge over 10 min under mooring conditions, which is slower than the horizontal misalignment angle (within 2 min only) and does not meet the demand for a quick start.

It is well known that the horizontal components of gravity projection in the horizontal coordinate frame are zero and the horizontal alignment is rapid. If an accurate horizontal coordinate frame is established, the interference caused by sway can be easily isolated. Based on this idea, we proposed an improved alignment scheme in [9]. Then, according to the characteristics of system structure, we used a dimensionality reduction Gauss-Hermite filter (GHF) algorithm to establish the accurate horizontal coordinate frame [17]. We specify the details of the algorithm and supplement error analysis in this paper. Because of the low precision of the traditional inertial frame alignment algorithm in the mooring environment, it is usually employed as a coarse alignment for a marine SINS. We use a clever method to improve the traditional inertial alignment algorithm in this paper, and improve the performance of the traditional inertial frame alignment algorithm.

Compared with the commonly used nonlinear filtering algorithms such as EKF [18,19], UKF [20] and CKF [21,22], GHQF [23] has the advantages of incomparable precision and stability. However, this approach is infeasible for high-dimensional systems since the computation burden increases exponentially with the index of dimension. This results in the “curse of dimensionality”. Fortunately, only misalignment angles suffer from nonlinearity in initial alignment, so that we can apply a dimension reduction nonlinear filter to carry out the alignment.

The remainder of this paper is organized as follows. Firstly, reference frames and parameter definitions are addressed in Section 2. The algorithmic principle for traditional alignment in the inertial frame is presented in Section 3. Section 4 details how to establish an accurate horizontal reference frame and Section 5 details how to accomplish the alignment. Then, the simulation results that validate the proposed approach are presented in Section 6. Section 7 presents the experimental results. Finally, the conclusions are presented in Section 8.

## 2. Reference Frames and Parameter Definitions

The reference frames are defined in Table 1 and the parameters are defined in Table 2.

**Table 1:** The reference frames definitions used in this paper.

Reference Frame	Definition
*n*-frame	Navigation reference frame which is the local horizontal reference frame. Its axes are aligned with east–north–up (ENU) geodetic axes.
*h*-frame	Horizontal reference frame. Its $z_{h}$ axis is aligned with $z_{n}$ axis, but the horizontal axes are arbitrary in the horizontal plane.
*e*-frame	Earth-centered Earth-fixed orthogonal reference frame. Its x_e_ axis points to the local longitude.
*b*-frame	Body reference frame aligned with inertial measurement unit (IMU) axes.
*i*-frame	Earth-centered inertial fixed (ECIF) orthogonal reference frame. The axes are fixed with *e*-frame at the beginning of the alignment process.
$i_{b0}$-frame	Body inertial reference frame. It is formed by fixing the axes of *b*-frame in the inertial space at the beginning of the alignment process.

**Figure 1:**
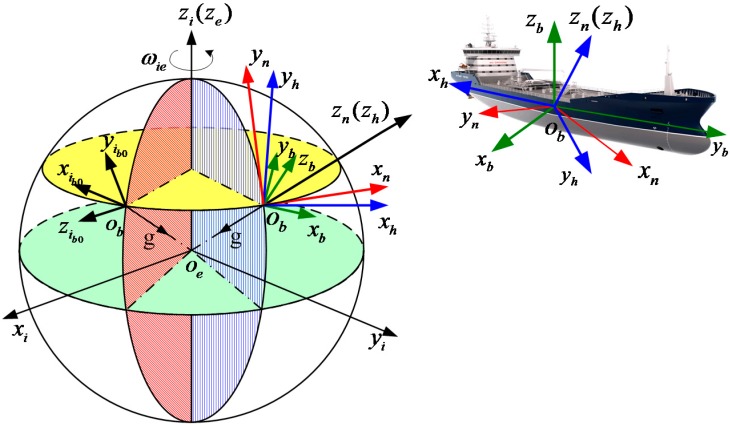
The relationship of the frames.

The relationship of the frames mentioned in Table 1 is shown in Figure 1.

**Table 2:** The parameters.

Parameter	Definition
$C_{p1}^{p2}$	Transform matrix from p1 frame to p2 frame
$\omega_{ie}$	Angular rate of Earth rotation
g	Gravity
L	Latitude
V	Velocity
ε	Gyro drift
∇	Acceleration bias
ϕ	Misalignment angle
f	Specific Force

## 3. The Algorithmic Scheme for an Alignment Algorithm in an Inertial Frame

The alignment algorithm in an inertial frame is based on the consideration that the Earth rate is constant in a body inertial reference frame, and we can get north from the projection of the gravity in the inertial reference frame which defines a cone whose main axis is the rotational axis of the Earth [3,7].

The traditional alignment algorithm in an inertial frame is presented in [3,4,5,7]. It is usually decomposing the strapdown matrix $C_{b}^{n}(t)$ (which represents the orientation of the *b* frame relative to the n frame) as per Equation (1):

(1) $$C_{b}^{n}(t)=C_{e}^{n}C_{i}^{e}(t)C_{i_{b0}}^{i}C_{b}^{i_{b0}}(t)$$

Under mooring conditions, $C_{e}^{n}$ is a function of latitude L, and $C_{i}^{e}(t)$ is a function of time t.

(2) $$C_{e}^{n}=\left[\begin{array}{ccc} 0 & 1 & 0\\ -\sin L & 0 & \cos L\\ \cos L & 0 & \sin L \end{array}\right]$$

(3) $$C_{i}^{e}(t)=\left[\begin{array}{ccc} \cos(\omega_{ie}t) & \sin(\omega_{ie}t) & 0\\ -\sin(\omega_{ie}t) & \cos(\omega_{ie}t) & 0\\ 0 & 0 & 1 \end{array}\right]$$

where $C_{b}^{i_{b0}}(t)$ can be updated by the gyro output (its initial value is a unit matrix):

(4) $${\dot{\text{C}}}_{b}^{i_{b0}}\left(t\right)=C_{b}^{i_{b0}}\left(t\right)\left[\left(\omega_{i_{b0}}^{b}\right)\times \right]$$

where $\left[\left(\omega_{i_{b0}}^{b}\right)\times \right]$ is the skew symmetric matrix of the vector $\omega_{i_{b0}}^{b}$ measured by the gyroscopes representing the angular rate of b -frame with respect to $i_{b0}$-frame, and $C_{i_{b0}}^{i}$ is calculated as:

(5) $$C_{i_{b0}}^{i}={\left[\begin{array}{c} {\left[g^{i}(t_{1})\right]}^{T}\\ {\left[g^{i}(t_{2})\right]}^{T}\\ {\left[g^{i}(t_{1})\times g^{i}(t_{2})\right]}^{T} \end{array}\right]}^{-1}\left[\begin{array}{c} {\left[g^{i_{b0}}(t_{1})\right]}^{T}\\ {\left[g^{i_{b0}}(t_{2})\right]}^{T}\\ {\left[g^{i_{b0}}(t_{1})\times g^{i_{b0}}(t_{2})\right]}^{T} \end{array}\right]$$

where, $g^{i}$ and $g^{i_{b0}}$ represent the projections of the gravity in the i frame and the $i_{b0}$ frame, respectively. Since $g^{n}$ is known, the projection in the inertial frame can be calculated as:

(6) $$g^{i}=C_{e}^{i}(t)C_{n}^{e}g^{n}$$

In order to restrain the interference of disturbing acceleration, we usually use the following equation instead of Equation (5):

(7) $$C_{i_{b0}}^{i}={\left[\begin{array}{c} {\left[V^{i}(t_{1})\right]}^{T}\\ {\left[V^{i}(t_{2})\right]}^{T}\\ {\left[V^{i}(t_{1})\times V^{i}(t_{2})\right]}^{T} \end{array}\right]}^{-1}\left[\begin{array}{c} {\left[V^{i_{b0}}(t_{1})\right]}^{T}\\ {\left[V^{i_{b0}}(t_{2})\right]}^{T}\\ {\left[V^{i_{b0}}(t_{1})\times V^{i_{b0}}(t_{2})\right]}^{T} \end{array}\right]$$

where $V^{i}(t_{j})=\int g^{i}(t_{j})dt$, $V^{i_{b0}}(t_{j})=\int g^{i_{b0}}(t_{j})dt\text{=}\int {\hat{\text{C}}}_{b}^{i_{b0}}f^{b}(t_{j})dt$, (j=1,2).

Taking into account that $C_{e}^{n}$, $C_{i}^{e}(t)$, $C_{b}^{i_{b0}}(t)$, and $g^{i}$ can all be calculated by the parameters known or measured, how to extract the pure gravity projection in the $i_{b0}$ frame from the output of accelerometers is the essential operation we have to carry out.

**Figure 2:**
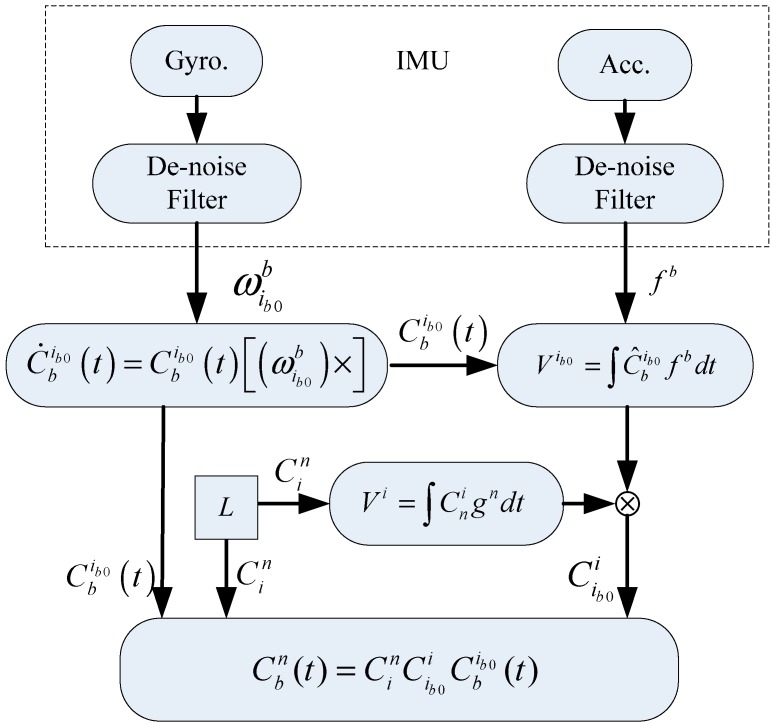
Flow chart of alignment algorithm in an inertial frame.

Figure 2 illustrates the algorithmic scheme.

## 4. The Establishment of a Horizontal Reference Frame

In Appendix A, we analyze the propagation of errors, and find it has the same precision as other methods. Therefore, if we can isolate the interference errors, the inertial frame alignment algorithm can be used for fine alignment.

In Section 3, we have pointed out that the essential and difficult process is to extract the pure gravity from measuring signals which are often interfered with by environmental disturbance. In order to obtain the accurate gravity projection in the $i_{b0}$ frame, we proposed a clever solution by using a feature of gravity. It is easy to obtain the pure gravity once an accurate horizontal reference frame is established, since the projection of gravity in the *h*-frame has nothing to do with the heading, and it is also relatively easy to establish the *h*-frame. In this section, we will show how to establish an accurate horizontal reference frame.

### 4.1. Nonlinear Error Model of a SINS

Traditional linear differential equations are based on the assumption that the misalignment angles are small. However, for a small misalignment angle model, a coarse alignment is necessary. To improve the accuracy and reduce the time, the nonlinear error model of large misalignment angle for a SINS described in [24] is adopted in this paper (see Section 2.1 in [24]).

Attitude error equation:

(8) $$\dot{\phi }=C_{\omega }^{-1}\left[\left(I-C_{n}^{n^{\prime }}\right){\hat{\omega }}_{in}^{n}+C_{n}^{n^{\prime }}\delta \omega_{in}^{n}-C_{b}^{n^{\prime }}\delta \omega_{ib}^{b}\right]$$

Velocity error equation:

(9) $$\delta {\dot{\text{v}}}^{n}=\left[I-{\left(C_{n}^{n^{\prime }}\right)}^{T}\right]C_{b}^{n^{\prime }}{\hat{\text{f}}}^{b}+C_{b}^{n}\nabla^{b}-\left(2\delta \omega_{ie}^{n}+\delta \omega_{en}^{n}\right)\times \left({\hat{v}}^{n}-\delta v^{n}\right)-\left(2{\hat{\omega }}_{ie}^{n}+{\hat{\omega }}_{en}^{n}\right)\times \delta v^{n}+C_{b}^{n}w_{a}^{b}$$

Position error equations:

(10) $$\{\begin{array}{c} \delta \dot{\text{L}}=\frac{\delta v_{N}^{n}}{R_{M}}\\ \delta \dot{\lambda }=\frac{\sec L}{R_{N}}\delta v_{E}^{n}+\frac{v_{E}^{n}\sec L\tan L}{R_{N}}\delta L \end{array}$$

where superscript $n^{\prime }$ donates the calculation navigation reference frame, and $\phi ={\left[\begin{array}{ccc} \phi_{x} & \phi_{y} & \phi_{z} \end{array}\right]}^{T}$ is the Euler error angle vector. $c(\phi_{i})$ and $s(\phi_{i})$ denote $\cos\phi_{i}$ and $\sin\phi_{i}$, respectively.

(11) $$C_{n}^{n^{\prime }}=\left[\begin{array}{ccc} c(\phi_{y})c(\phi_{z})-s(\phi_{y})s(\phi_{x})s(\phi_{z}) & c(\phi_{y})s(\phi_{z})+s(\phi_{y})s(\phi_{x})c(\phi_{z}) & -s(\phi_{y})c(\phi_{x})\\ -c(\phi_{x})s(\phi_{z}) & c(\phi_{x})c(\phi_{z}) & s(\phi_{x})\\ s(\phi_{y})c(\phi_{z})+c(\phi_{x})s(\phi_{x})s(\phi_{z}) & s(\phi_{y})s(\phi_{z})-c(\phi_{y})s(\phi_{x})c(\phi_{z}) & c(\phi_{x})c(\phi_{y}) \end{array}\right]$$

(12) $$C_{\omega }=\left[\begin{array}{ccc} c(\phi_{y}) & 0 & -s(\phi_{y})c(\phi_{x})\\ 0 & 1 & s(\phi_{x})\\ s(\phi_{y}) & 0 & c(\phi_{y})c(\phi_{x}) \end{array}\right]$$

(13) $${\hat{\omega }}_{in}^{n}={\hat{\omega }}_{ie}^{n}+{\hat{\omega }}_{en}^{n}$$

(14) $${\delta \omega }_{in}^{n}={\delta \omega }_{ie}^{n}+{\delta \omega }_{en}^{n}$$

(15) $${\delta \omega }_{ib}^{b}=\varepsilon^{b}+w_{g}^{b}$$

where ${\hat{\omega }}_{ie}^{n}$ is the calculated Earth’s rotating angular rate, ${\hat{\omega }}_{en}^{n}$ is the calculated angular rate vector, and ${\delta \omega }_{in}^{n}$ is the calculated error vector of $\omega_{in}^{n}$. ${\delta \omega }_{ie}^{n}$ and ${\delta \omega }_{en}^{n}$ are respectively the error vectors of ${\hat{\omega }}_{ie}^{n}$ and ${\hat{\omega }}_{en}^{n}$ $\varepsilon^{b}$ and $w_{g}^{b}$ are the gyro constant drift vector and the zero-mean Gaussian white noise vector, respectively. ${\hat{f}}^{b}$ and $\delta f^{b}$ denote the specific force vector and its corresponding error vector, respectively. ${\hat{v}}^{n}$ and $\delta v^{n}$ are calculated velocity vector and its corresponding error vector, respectively. $R_{M}$ and $R_{N}$ are the Earth’s radii of the meridian circle and the prime vertical circle, respectively.

### 4.2. The Dimension Reduction Gauss-Hermite Filter

The Gauss-Hermite filter (GHF) is one of the sigma point filters. It has proved to be efficient and successful in solving estimation problems when the state and noise distributions are Gaussian. It is usually used as a benchmark algorithm, since its accuracy and stability are the highest among numerous Gaussian approximation filters [23,25,26] (the algorithm framework see Appendix B). However, the “curse of dimensionality” would seriously affect the real-time performance for high dimensional systems [23,27]. From Section 4.1, it is known that only misalignment angle suffers from nonlinearity in the nonlinear error model. This means it is possible to employ a dimension reduction GHF to deal with the alignment task.

In order to establish an accurate horizontal reference frame and reduce the amount of calculation, we employ the dimension reduction GHF algorithm.

The large misalignment angle error model of SINS alignment is a typical nonlinear model that can be described as a general form:

(16) $$x_{k+1}=F_{k}(\xi_{k})x_{k}+g_{k}(\xi_{k})+w_{k}$$

(17) $$y_{k}=H_{k}(\varsigma_{k})x_{k}+h_{k}(\varsigma_{k})+v_{k}$$

where $\xi_{k}$ is the first l components of $x_{k}$, $\varsigma_{k}$ is arbitrary s components of $x_{k}$.

**Figure 3:**
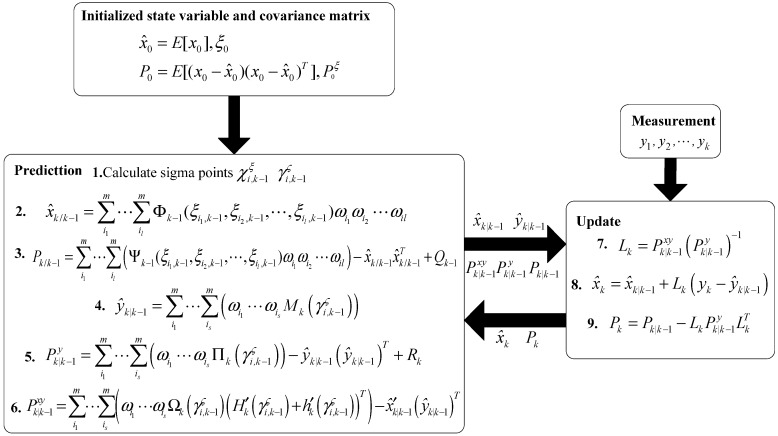
The flow chart of the dimension reduction GHF.

The dimension reduction GHF algorithm is shown in Figure 3.

where,

(18) $$\Phi_{k-1}(\xi_{k-1})=F_{k-1}(\xi_{k-1})\left({\hat{x}}_{k-1}+S_{k-1}\left[\begin{array}{c} {\left(S_{k-1}^{\xi }\right)}^{-1}(\xi_{k-1}-{\hat{\xi }}_{k-1})\\ 0 \end{array}\right]\right)+g_{k-1}(\xi_{k-1})$$

(19) $$\Psi_{k-1}(\xi_{k-1})=\Phi_{k-1}(\xi_{k-1})\Phi_{k-1}^{T}(\xi_{k-1})+F_{k-1}(\xi_{k-1})S_{k-1}\left[\begin{array}{cc} 0 & 0\\ 0 & I_{n-l} \end{array}\right]S_{k-1}^{T}F_{k-1}^{T}(\xi_{k-1})$$

(20) $$\Theta_{k}(\varsigma_{k})={{\hat{x}}^{\prime }}_{k/k-1}+S_{k/k-1}\left[\begin{array}{c} {\left(S_{k/k-1}^{\varsigma }\right)}^{-1}(\varsigma_{k}-{\hat{\varsigma }}_{k/k-1})\\ 0 \end{array}\right]$$

(21) $$\Omega_{k}(\varsigma_{k})=\Theta_{k}(\varsigma_{k})\Theta_{k}^{T}(\varsigma_{k})+S_{k/k-1}\left[\begin{array}{cc} 0 & 0\\ 0 & I_{t} \end{array}\right]S_{k/k-1}^{T}$$

(22) $${Μ}_{k}(\varsigma_{k})={H^{\prime }}_{k}(\varsigma_{k})\Theta_{k}(\varsigma_{k})+h_{k}(\varsigma_{k})$$

(23) $$\Pi_{k}(\varsigma_{k})={Μ}_{k}^{T}(\varsigma_{k}){Μ}_{k}^{T}(\varsigma_{k})+{H^{\prime }}_{k}(\varsigma_{k}){S^{\prime }}_{k/k-1}\left[\begin{array}{cc} 0 & 0\\ 0 & I_{t} \end{array}\right]{\left({S^{\prime }}_{k/k-1}\right)}^{T}F^{T}(\varsigma_{k})$$

where, $P^{\xi }$ denotes the first l-th rows and the first l-th columns of the matrix P; $S^{\xi }$ and S are obtained from $P^{\xi }$ and P, respectively, through a Cholesky decomposition; that is $P=SS^{T}$, $P^{\xi }=S^{\xi }{\left(S^{\xi }\right)}^{T}$. $\hat{\xi }$ denotes the first l-th components of $\hat{x}$, and m=n−l. The quadrature points $\left\{\xi_{i}\right\}$ and the associated weights $\left\{\omega_{i}\right\}$ are determined by the Gauss–Hermite quadrature rule (see Appendix C).

### 4.3. The Horizontal Alignment for the Large Misalignment Angle Model Based on the Dimension Reduction Gauss-Hermite Filter

As mentioned above, the nonlinear model for horizontal alignment is established under the large misalignment angle in this paper. Considering 13 state variables—the east velocity error $\delta v_{E}$ and the north velocity erro $\delta v_{N}$; the Euler misalignment angle errors $\phi_{x}$ $\phi_{y}$ and $\phi_{z}$; the latitude error δL and the longitude erro δλ; the accelerometer zero-biases $\nabla_{x}^{b}$ $\nabla_{y}^{b}$ and $\nabla_{z}^{b}$; the constant gyro drifts $\varepsilon_{x}^{b}$ $\varepsilon_{y}^{b}$ and $\varepsilon_{z}^{b}$—the state vector is built up as

$$X={\left[\begin{array}{ccccccccccccc} \delta v_{E} & \delta v_{N} & \phi_{x} & \phi_{y} & \phi_{z} & \delta L & \delta \lambda & \nabla_{x}^{b} & \nabla_{y}^{b} & \nabla_{z}^{b} & \varepsilon_{x}^{b} & \varepsilon_{y}^{b} & \varepsilon_{z}^{b} \end{array}\right]}^{T}$$

The corresponding state equation is written as:

$${\dot{\text{X}}}_{k+1}=f(X_{k})+W_{k}$$

The state function f(·) is obtained from Equations (8)–(10).

We choose the quadrature point m=3, then the total number of points $N_{p}=3^{13}$, and that is a great amount of computation. However, according to Section 4.1, we know that only the Euler misalignment angle errors $\phi_{x}$, $\phi_{y}$, and $\phi_{z}$ are suffering from nonlinearity; therefore, the dimension reduction Gauss-Hermite filter proposed in Section 4.2. can be adopted, and the number of points will be reduced to $N_{p}=3^{3}$. That greatly reduces the computational burden.

## 5. Calculation of the Gravity Direction

In Section 5, we present a method to set-up an accuracy horizontal reference frame in detail. After the fine alignment, we obtain the transfer matrix $C_{b}^{h}$, that is to say, the accuracy horizontal reference frame is established. Then, the projection of the gravity in the $i_{b0}$ frame can be calculated as:

(24) $$g^{i_{b0}}(t)=C_{b}^{i_{b0}}(t){\left[C_{b}^{h}\right]}^{-1}g^{h}$$

where, $g^{h}=g^{n}={\left[\begin{array}{ccc} 0 & 0 & g \end{array}\right]}^{T}$, $C_{b}^{i_{b0}}(t)$ is updated by the gyros’ output in real time, and $C_{b}^{h}$ is obtained from the fine horizontal alignment.

In order to improve the accuracy further, a weighted smoothing algorithm is adopted to inhibit the interference noise caused by the winds and waves. The algorithm is described as follows:

Assume that $t_{i}(i\in (1,N))$ is the sampling period, $g_{i}$ is the corresponding sampling data, and the weight coefficient is 1. Then, the smoothed data is calculated as:

(25) $$\bar{g}=\frac{1}{N+1}\sum_{i=-N/2}^{N/2}g_{i}$$

**Figure 4:**
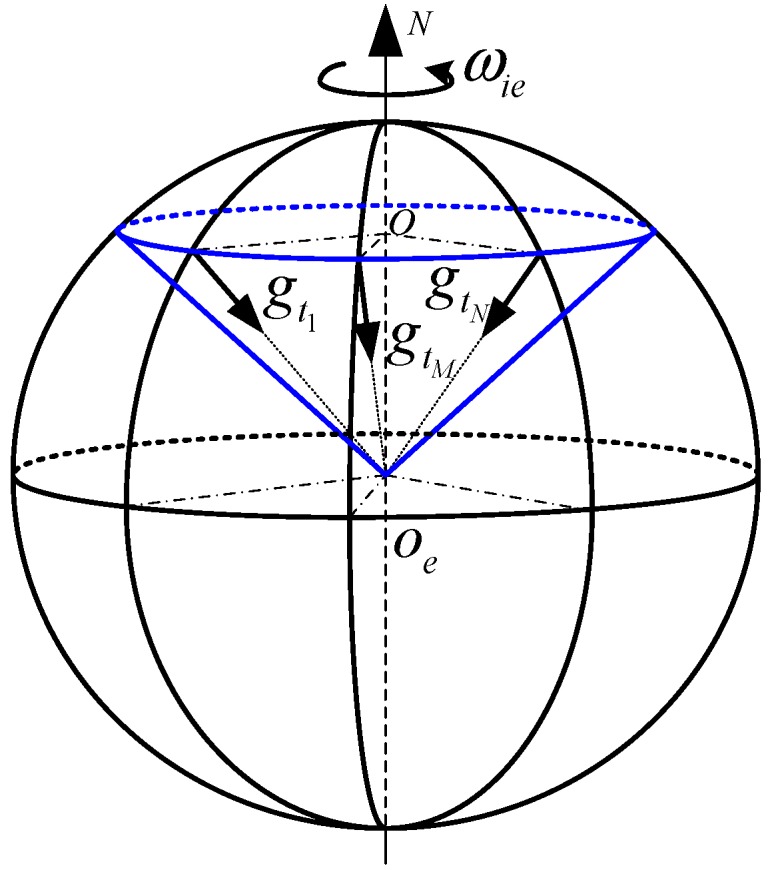
The coning motion of gravity.

Since it is a coning motion of gravity in an inertial frame ($i_{b0}$ frame or i frame), as Figure 4, the projection of $g^{i_{b0}}$ in the $i_{b0}$ frame is a sine curve, and the period is 24 h. Compared with the alignment time, the period is so long that we can consider $g^{i_{b0}}(t)$ as linear g.

Then,

(26) $$g_{N}^{i_{b0}}=C_{t_{M}}^{t_{N}}\bar{g}$$

where the transfer matrix from $n_{M}$ frame (at $t_{M}$) to n frame (at $t_{N}$) could be calculated as follows:

(27) $$C_{t_{M}}^{t_{N}}=C_{b}^{ib0}(t_{N})\cdot {\left[C_{b}^{ib0}(t_{M})\right]}^{-1}$$

We can smooth $g^{i_{b0}}$ at 1 and 5 min respectively with Equation (25) after the fine horizontal alignment, and then $C_{i}^{i_{b0}}$ can be calculated with Equation (4).

Finally, by substituting $C_{i}^{i_{b0}}$ into Equation (1), the alignment can be completed.

**Figure 5:**
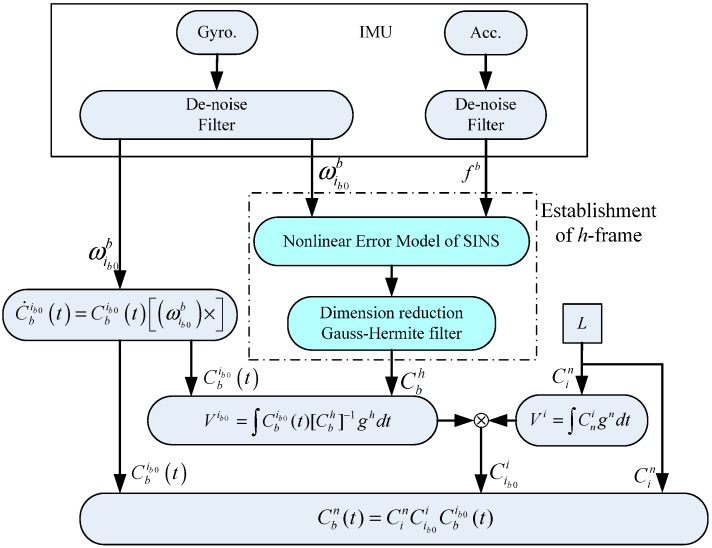
Flow chart of the improved alignment algorithm.

The flow chart of the improved alignment algorithm is shown in Figure 5.

As shown in the dashed box, we use a clever method to construct matrix $V^{i_{b0}}$. This improvement makes the traditional inertial alignment algorithm able to resist interference.

## 6. Simulation

In order to test the technique proposed in this paper, a simulation is carried out to compare with the conventional inertial frame alignment algorithm. The main parameters are set as follows in Table 3:

**Table 3:** Specifications of IMU.

Parameters	Gyro	Accelerometer
Constant bias	0.01°/h	$10^{-4}g$
Random noise	0.05°$\sqrt{\text{h}}$	$0.5\times 10^{-4}g/\sqrt{\text{Hz}}$

(1) The latitude and longitude: L=45.7796°, λ=126.6705°;

(2) The misalignment angle: $\phi_{x}=10$°, $\phi_{y}=10$°, $\phi_{z}=10$°;

(3) The sampling period is 0.1 s;

(4) The initial attitude is a random value.

(5) The first sampling time is $t_{k1}=70$ s, and the second is $t_{k2}=300$ s.

(6) We set up two situations to test the proposed algorithm, and the model is as follows:

Situation 1: Assume the ship is in the state of rest, without any interference.

Situation 2: Assume that the ship is on the berth and is rocked by the surf and wind. The pitch, roll, and yaw models of a marine vehicle are given by:

(28) $$\psi =\psi_{0}+\psi_{m}\sin(2\pi t/T_{\psi })$$

(29) $$\theta =\theta_{0}+\theta_{m}\sin(2\pi t/T_{\theta })$$

(30) $$\gamma =\gamma_{0}+\gamma_{m}\sin(2\pi t/T_{\gamma })$$

where, ψ θ, and γ are yaw, pitch, and roll angles, respectively; the initial attitudes are $\psi_{0}$ = 30°, $\theta_{0}=\gamma_{0}=0$; the sway periods are $T_{\psi }=6$ s, $T_{\theta }=10$ s, and $T_{\gamma }=8$ s; $\psi_{m}$ = 1°, $\theta_{m}$ = 5°, and $\gamma_{m}$ = 5°.

In the mooring condition, the horizontal velocities are small values because the mooring line secures the ship to the wharf, but vertical velocity may be not as small as horizontal velocities since a ship will heave along with the sea level fluctuation. So, velocity interference models are given by:

(31) $$V_{dz}=0.5\cdot \sin(\omega_{dz}t+\varphi_{dz})$$

(32) $$V_{dx}=0.02\cdot \sin(\omega_{dx}t+\varphi_{dx})$$

(33) $$V_{dy}=0.02\cdot \sin(\omega_{dy}t+\varphi_{dy})$$

where $\omega_{d}=\frac{2\pi }{T_{d}}$; $T_{dz}=8$ s; $T_{dx}=T_{dy}=2$ s; $\varphi_{dx},\varphi_{dy},\varphi_{dz}$ are random values in [0,2π].

The parameters of the dimension reduction GHF are chosen as follows:

$$\begin{array}{c} {\hat{x}}_{0}=[\begin{array}{ccccccc} 10^{\text{o}} & 10^{\text{o}} & 10^{\text{o}} & 0.1\text{m/s} & 0.1\text{m/s} & 0.1\text{m} & 0.1\text{m} \end{array}\\ \begin{array}{cccccc} \begin{array}{cc} & 1\times 10^{-4}g_{0} \end{array} & 1\times 10^{-4}g_{0} & 1\times 10^{-4}g_{0} & 0.01^{\text{o}}\text{/h} & 0.01^{\text{o}}\text{/h} & 0.01^{\text{o}}\text{/h} \end{array}] \end{array}$$

$$\begin{array}{c} P_{0}=diag[\begin{array}{ccccccc} {\left(5^{\text{o}}\right)}^{2} & {\left(5^{\text{o}}\right)}^{2} & {\left(5^{\text{o}}\right)}^{2} & {\left(0.2\text{m/s}\right)}^{2} & {\left(0.2\text{m/s}\right)}^{2} & {\left(0.2\text{m}\right)}^{2} & {\left(0.2\text{m}\right)}^{2} \end{array}\\ \begin{array}{cccccc} {\left(1\times 10^{-4}g_{0}\right)}^{2} & {\left(1\times 10^{-4}g_{0}\right)}^{2} & {\left(1\times 10^{-4}g_{0}\right)}^{2} & {\left(0.01^{\text{o}}\text{/h}\right)}^{2} & {\left(0.01^{\text{o}}\text{/h}\right)}^{2} & {\left(0.01^{\text{o}}\text{/h}\right)}^{2} \end{array}] \end{array}$$

$$Q=diag\left[\begin{array}{ccccc} {\left(1\times 10^{-4}g_{0}\right)}^{2} & {\left(1\times 10^{-4}g_{0}\right)}^{2} & {\left(0.01^{\text{o}}\text{/h}\right)}^{2} & {\left(0.01^{\text{o}}\text{/h}\right)}^{2} & {\left(0.01^{\text{o}}\text{/h}\right)}^{2} \end{array}\right]$$

$$R=diag\left[\begin{array}{cc} {\left(0.01\text{m/s}\right)}^{2} & {\left(0.01\text{m/s}\right)}^{2} \end{array}\right]$$

For a fair comparison, 100 independent Monte Carlo runs are carried out. The results are as follows; the RMS error results are used to test the horizontal alignment accuracy and time consumed by dimensionality reduction GHF. RMS error is defined in Appendix D.

**Figure 6:**
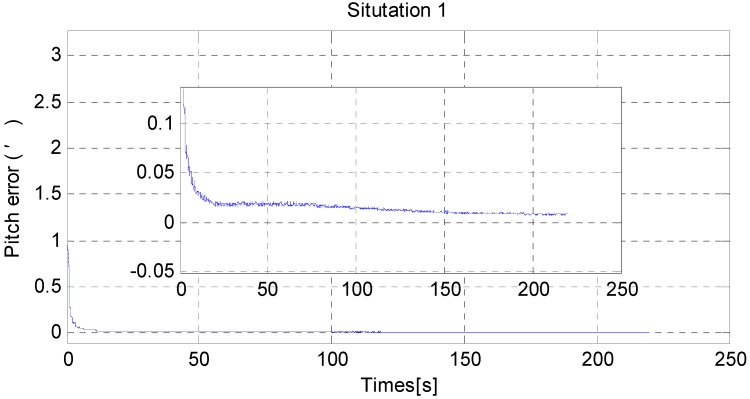
The RMS error of pitch misalignment angle in situation 1.

**Figure 7:**
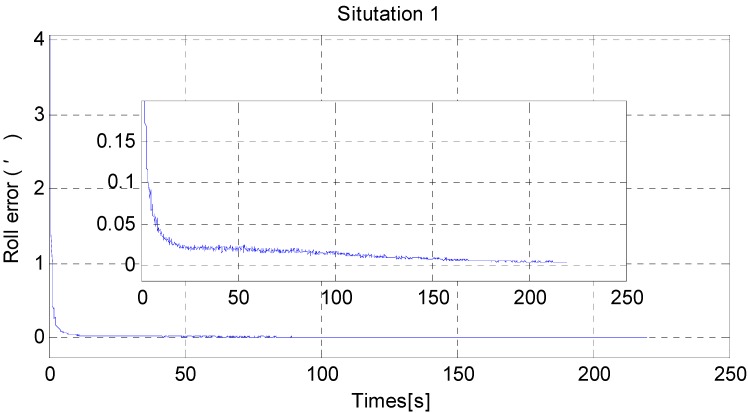
The RMS error of roll misalignment angle in situation 1.

**Figure 8:**
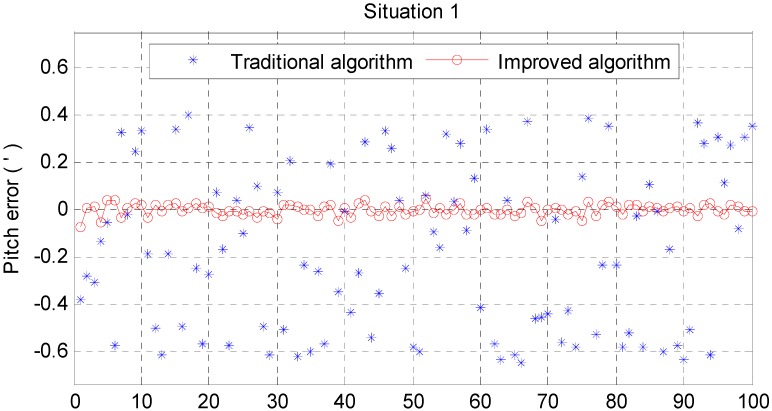
Pitch error result in situation 1.

**Figure 9:**
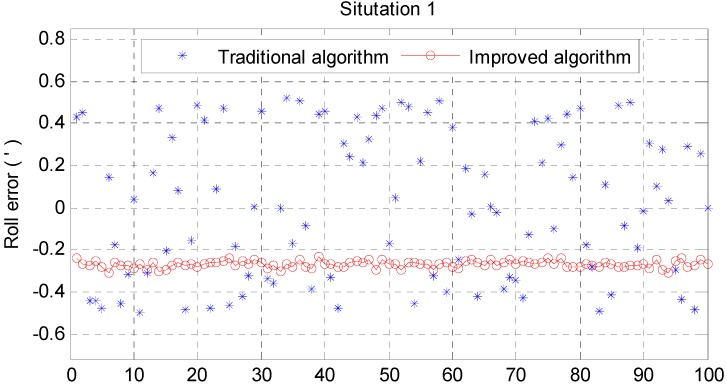
Roll error result in situation 1.

**Figure 10:**
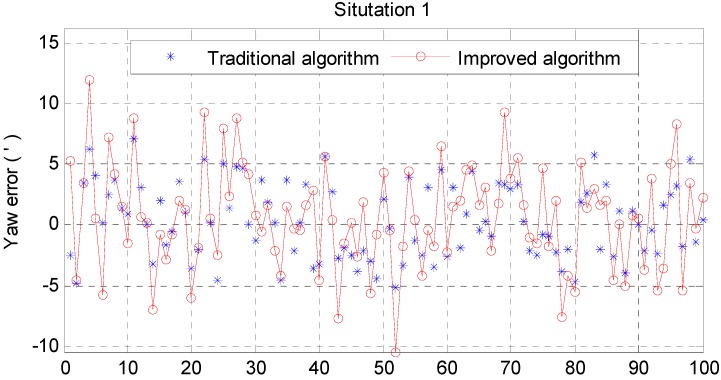
Yaw error result in situation 1.

From Figure 6 and Figure 7, it can be noted that the pitch error and roll error converge to the order of minutes within a few seconds. Figure 8, Figure 9 and Figure 10 show the pitch error, roll error, and yaw error calculated by the traditional algorithm and the proposed algorithm, respectively. Their statistical results are shown in Table 4. From Table 4, we can tell that the accuracy of heading alignment is roughly the same (mean value, standard deviation and maximum value). However, the horizontal alignment results from the proposed algorithm are obvious better, since the standard deviation and the maximum value are smaller; the standard deviation in particular is an order of magnitude smaller than the traditional algorithm.

**Table 4:** Statistics of situation 1.

	Traditional Algorithm (min)			Improved Algorithm (min)		
	Pitch Error	Roll Error	Yaw Error	Pitch Error	Roll Error	Yaw Error
Mean	−0.1677	0.0156	0.3089	−0.0001	−0.2679	0.6363
Std	0.3475	0.3467	3.0679	0.0231	0.0163	4.2559
Max	0.6472	0.5212	7.1117	0.0723	0.3059	11.9556

**Figure 11:**
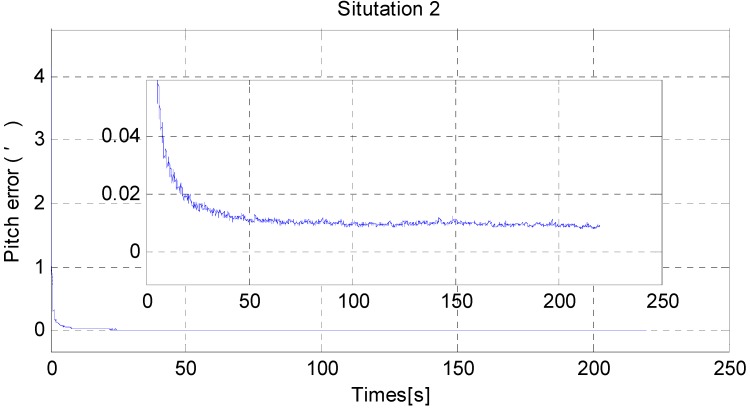
The RMS error of pitch misalignment angle in situation 2.

**Figure 12:**
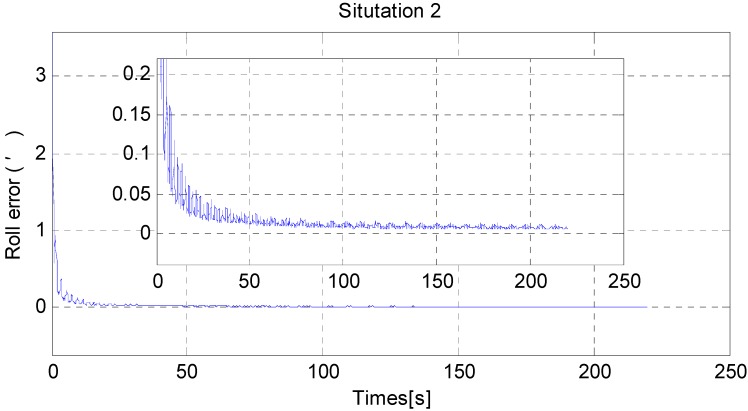
The RMS error of roll misalignment angle in situation 2

**Figure 13:**
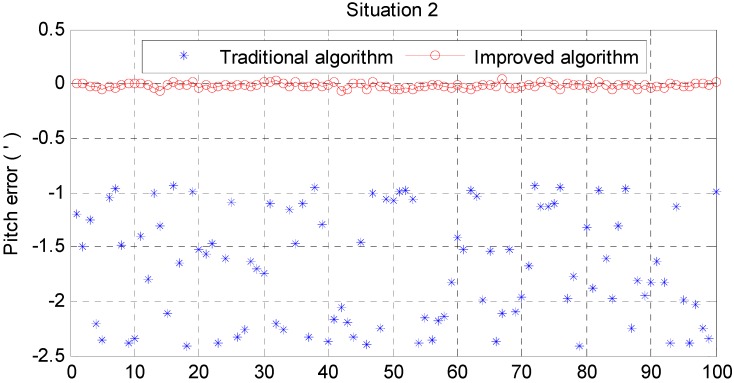
Pitch error result in situation 2.

**Figure 14:**
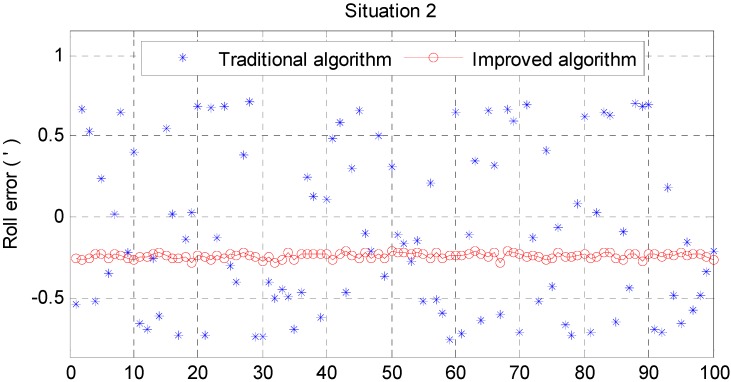
Roll error result in situation 2.

**Figure 15:**
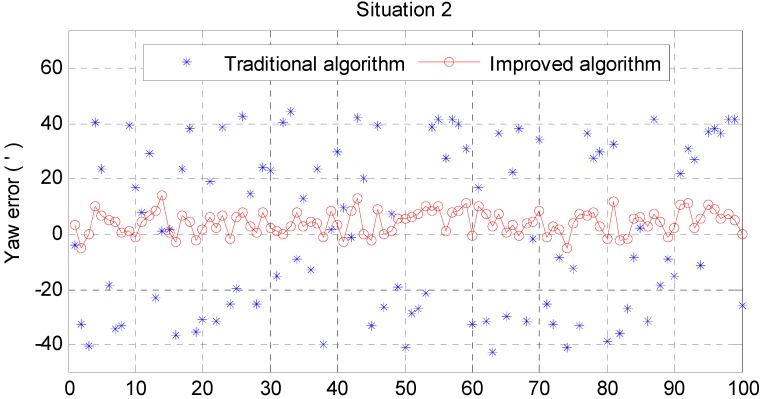
Yaw error result in situation 2.

Figure 11 and Figure 12 show the horizontal alignment results of the nonlinear filter in situation 2. The pitch error and roll error also converge rapidly (within tens of seconds) even though they converge slower than situation 1. Figure 13, Figure 14 and Figure 15 show the pitch error, roll error, and yaw error calculated by traditional algorithm and the proposed algorithm in situation 2, respectively. Their statistical results are shown in Table 5. From Table 5, we can draw the conclusion that the proposed algorithm performs much better than the traditional one, because not only is the horizontal alignment accuracy an order of magnitude higher than the traditional one’s (standard deviation and maximum value), but also the heading alignment accuracy of the proposed algorithm is better than the traditional one’s. Compared with Table 4 and Table 5, we can see that angular velocities and velocities have little effect on the proposed algorithm, but will greatly affect the precision of the traditional algorithm.

**Table 5:** Statistics of situation 2.

	Traditional Algorithm (angular minute)			Improved Algorithm (angular minute)		
	Pitch Error	Roll Error	Yaw Error	Pitch Error	Roll Error	Yaw Error
Mean	−1.6843	−0.0872	2.5054	−0.0172	−0.2397	4.1585
Std	0.5111	0.5033	29.3313	0.02248	0.0174	4.2575
Max	2.4103	0.7588	44.1109	0.0680	0.2863	14.1987

## 7. Experiments

**Figure 16:**
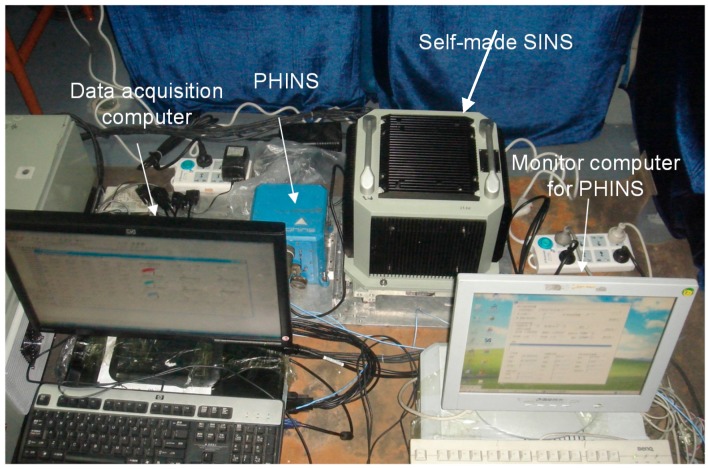
Self-made SINS, PHINS, and data acquisition computer.

In order to evaluate the performance of the proposed self-alignment method for SINS, in this section, the mooring experiment was conducted in the East Sea of China. In this experiment, the ship was moored to the pier. A self-made SINS was used for the experiment, and the attitude reference was given by a PHINS (made by iXBlue Company) as shown in Figure 16. The self-made SINS and the PHINS were fixed on a rigid aluminum alloy board, and then the installation error was measured and compensated for in the stationary state. The data acquisition computer collected the data of the self-made SINS and the PHINS synchronously. We carried out the alignment experiments three times.

The PHINS worked in GPS aided mode, its performance is as follows: pitch and roll errors are less than 0.01°, and heading error is less than 0.02°. In this experiment, we used the dimension reduction GHF mentioned in Section 4.2 to implement horizontal alignment. During the mooring experiments, the parameters of the dimension reduction GHF were optimally chosen as follows:

$$\begin{array}{c} {\hat{x}}_{0}=[\begin{array}{ccccccc} 0^{\text{o}} & 0^{\text{o}} & 0^{\text{o}} & 0\text{m/s} & 0\text{m/s} & 0\text{m} & 0\text{m} \end{array}\\ \begin{array}{cccccc} \begin{array}{cc} & 1\times 10^{-4}g_{0} \end{array} & 1\times 10^{-4}g_{0} & 1\times 10^{-4}g_{0} & 0.01^{\text{o}}\text{/h} & 0.01^{\text{o}}\text{/h} & 0.01^{\text{o}}\text{/h} \end{array}] \end{array}$$

$$\begin{array}{c} P_{0}=diag[\begin{array}{ccccccc} {\left(1^{\text{o}}\right)}^{2} & {\left(1^{\text{o}}\right)}^{2} & {\left(3^{\text{o}}\right)}^{2} & {\left(0.2\text{m/s}\right)}^{2} & {\left(0.2\text{m/s}\right)}^{2} & {\left(1\text{m}\right)}^{2} & {\left(1\text{m}\right)}^{2} \end{array}\\ \begin{array}{cccccc} {\left(1\times 10^{-4}g_{0}\right)}^{2} & {\left(1\times 10^{-4}g_{0}\right)}^{2} & {\left(1\times 10^{-4}g_{0}\right)}^{2} & {\left(0.01^{\text{o}}\text{/h}\right)}^{2} & {\left(0.01^{\text{o}}\text{/h}\right)}^{2} & {\left(0.01^{\text{o}}\text{/h}\right)}^{2} \end{array}] \end{array}$$

$$Q=diag\left[\begin{array}{ccccc} {\left(1\times 10^{-4}g_{0}\right)}^{2} & {\left(1\times 10^{-4}g_{0}\right)}^{2} & {\left(0.01^{\text{o}}\text{/h}\right)}^{2} & {\left(0.01^{\text{o}}\text{/h}\right)}^{2} & {\left(0.01^{\text{o}}\text{/h}\right)}^{2} \end{array}\right]$$

$$R=diag\left[\begin{array}{cc} {\left(0.1\text{m/s}\right)}^{2} & {\left(0.1\text{m/s}\right)}^{2} \end{array}\right]$$

**Figure 17:**
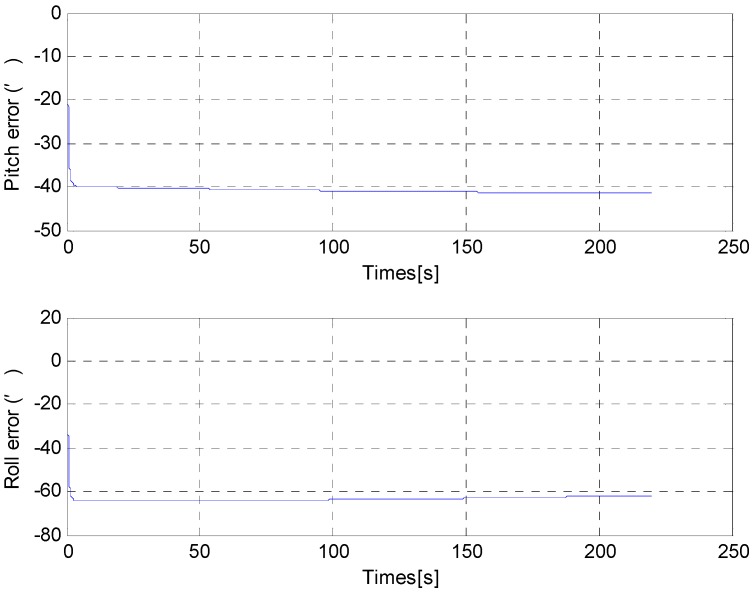
Estimations of misalignment angle.

The results are shown as follows. Figure 17 shows the estimations of misalignment angle.

**Figure 18:**
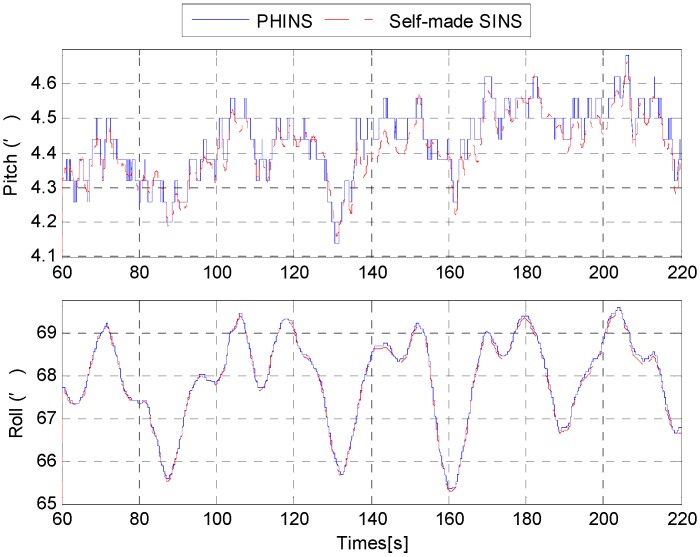
Attitude of PHINS and self-made SINS.

**Figure 19:**
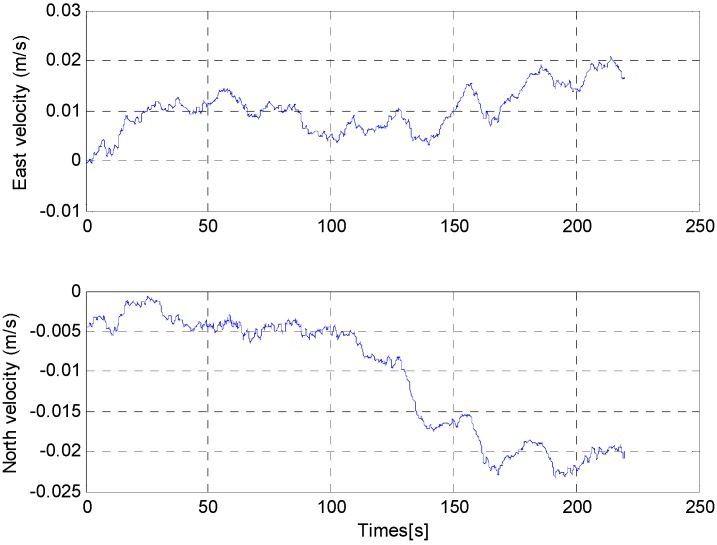
Velocity of PHINS.

From Figure 17, the estimations of misalignment angle converge within a few seconds. In order to ensure the estimations of misalignment angle are available, the pitch error and roll error are compensated for after 60 s. Figure 18 shows the attitude of the PHINS and the self-made SINS. For convenience, the initial 60 s part was omitted. We note that the difference between the two curves is very small (less than 0.05′). This indicates that the accurate horizontal reference frame is already established successfully. It is also proved that the estimations of the pitch error and roll error are accurate. From the attitude curves in Figure 18 and the velocity curves in Figure 19, we can also find that there are periodic disturbances during the alignment process.

**Figure 20:**
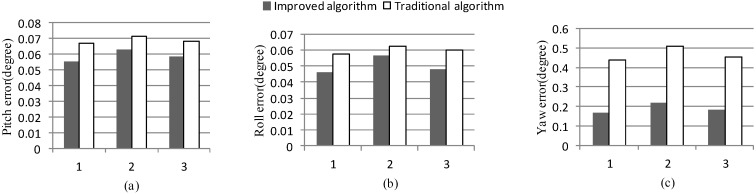
Comparison of the misalignment angles. (**a**) Pitch error; (**b**) roll error; (**c**) yaw error.

Figure 20 presents the alignment results for the 3-time experiment. It is clear from Figure 20 that the alignment results of the improved algorithm are better than the traditional algorithm. The pitch errors and the roll errors of the improved algorithm are less than 0.06°, and yaw errors are less than 0.2°. That meets the accuracy requirements of a medium-accuracy marine SINS.

## 8. Conclusions

An improved inertial frame alignment algorithm based on horizontal alignment for marine SINS is proposed in this paper. The major improvement of this work is establishing a horizontal reference frame using a dimension reduction Gauss-Hermite filter. Based on that, the projection of gravity in the body inertial reference frame can be calculated and take the place of the accelerometer output to calculate the attitude matrix.

The dimension reduction Gauss-Hermite filter algorithm is detailed in this paper. The simulation and experimental results indicate that it can quickly and accurately complete the horizontal alignment. However, the parameters *P_0_*, *Q*, and *R* which affect the filtering performance are usually chosen according to prior knowledge, as we have not yet found a mathematical method to get the optimal parameters. This means the selection of *P_0_*, *Q*, and *R* is rather a matter of tuning. We also derive the error propagation equation and point out that the inertial frame alignment algorithm has the same theoretical accuracy as other algorithms.

The results of the simulation and the experiment also show that, compared with the traditional inertial frame alignment algorithm, the proposed algorithm can resist velocity and angular velocity interference to obtain higher accuracy, and meets the requirement of a medium-accuracy inertial navigation system.

## Appendix

### A. Error Analysis

From Equation (1), the errors of the strapdown matrix $C_{b}^{n}(t)$ can be expressed as:

(A1) $$\delta {\text{C}}_{b}^{n}=\delta C_{e}^{n}C_{i}^{e}(t)C_{i_{b0}}^{i}C_{b}^{i_{b0}}(t)+C_{e}^{n}\delta C_{i}^{e}(t)C_{i_{b0}}^{i}C_{b}^{i_{b0}}(t)+C_{e}^{n}C_{i}^{e}(t)\delta C_{i_{b0}}^{i}C_{b}^{i_{b0}}(t)+C_{e}^{n}C_{i}^{e}(t)C_{i_{b0}}^{i}\delta C_{b}^{i_{b0}}(t)$$

Because Equations (2) and (3) are respectively the functions of latitude and time, and they are exactly known during the alignment process, so that $\delta C_{e}^{n}$ and $\delta C_{i}^{e}(t)$ can be ignored, the alignment error is caused by $\delta C_{i_{b0}}^{i}$ and $\delta C_{b}^{i_{b0}}(t)$.

Firstly, let us analyze the error of $C_{b}^{i_{b0}}(t)$. Its initial value is a constant matrix and contains no errors, so we only need to consider its updating error. In practice, $C_{b}^{i_{b0}}(t)$ is calculated through a differential equation:

(A2) $${\dot{\hat{\text{C}}}}_{b}^{i_{b0}}={\hat{\text{C}}}_{b}^{i_{b0}}\left({\hat{\omega }}_{ib}^{b}\times \right)$$

where $\left({\hat{\omega }}_{ib}^{b}\times \right)$ is the skew symmetric matrix of the vector; ${\hat{\omega }}_{ib}^{b}\text{=}{\left[\begin{array}{ccc} {\hat{\omega }}_{ibx}^{b} & {\hat{\omega }}_{iby}^{b} & {\hat{\omega }}_{ibz}^{b} \end{array}\right]}^{T}$ which is measured by the gyros, represents the turn rate of the b frame with respect to the i frame; and ${\hat{\omega }}_{ib}^{b}{=\omega }_{ib}^{b}{+\varepsilon }^{b}$, $\varepsilon^{b}$ is the gyro bias. Obviously, $\varepsilon^{b}$ directly produces the error in the calculation of $C_{b}^{i_{b0}}(t)$. We note that to solve Equation (7) we must carry out two integrations of $\varepsilon^{b}$, which will certainly increase the calculation error.

In order to facilitate analysis, we define ϕ as the attitude error angle,

(A3) $${\hat{C}}_{b}^{n}=\left(I-\left[\left(\phi^{n}\right)\times \right]\right)C_{b}^{n}$$

(A4) $${\hat{C}}_{i_{b0}}^{i}=\left(I-\left[\left(\phi^{i}\right)\times \right]\right)C_{i_{b0}}^{i}$$

(A5) $${\hat{C}}_{b}^{i_{b0}}=\left(I-\left[\left(\phi^{i_{b0}}\right)\times \right]\right)C_{b}^{i_{b0}}$$

Substitute Equations (A3)–(A5) into Equation (1), and ignore the second-order small items. We get:

(A6) $$\begin{array}{c} {\hat{\text{C}}}_{b}^{n}=C_{i}^{n}{\hat{\text{C}}}_{i_{b0}}^{i}{\hat{\text{C}}}_{b}^{i_{b0}}\\ =C_{i}^{n}\left(I-\left[\left(\phi^{i}\right)\times \right]\right)C_{i_{b0}}^{i}\left(I-\left[\left(\phi^{i_{b0}}\right)\times \right]\right)C_{b}^{i_{b0}}\\ \approx C_{i}^{n}\left(C_{b}^{i}-C_{i_{b0}}^{i}\left[\left(\phi^{i_{b0}}\right)\times \right]C_{i}^{i_{b0}}C_{b}^{i}-\left[\left(\phi^{i}\right)\times \right]C_{b}^{i}\right)\\ \text{=}\left(I-\left[\left(\phi_{2}^{n}\right)\times \right]-\left[\left(\phi_{3}^{n}\right)\times \right]\right)C_{b}^{n} \end{array}$$

where the similarity transformation theorem for matrix is used, and

(A7) $$\phi_{\text{2}}^{n}=C_{i_{b0}}^{n}\phi^{i_{b0}}$$

(A8) $$\phi_{\text{3}}^{n}=C_{i}^{n}\phi^{i}$$

Comparing Equation (A3) and Equation (A6), we know that:

(A9) $$\phi^{n}=\phi_{\text{2}}^{n}+\phi_{\text{3}}^{n}$$

Considering that ${\hat{\omega }}_{i_{b0}}^{b}=\omega_{i_{b0}}^{b}+\varepsilon^{b}$, $\varepsilon^{b}$ is gyros’ bias, substitute Equation (A5) into Equation (4) and ignore the second-order small items, and we can obtain:

(A10) $${\dot{\phi }}^{i_{b0}}=-C_{b}^{i_{b0}}\varepsilon^{b}$$

By solving Equation (17), we get:

(A11) $$\phi^{i_{b0}}\left(t\right)=-\int_{0}^{t}C_{b}^{i_{b0}}\left(t\right)\varepsilon^{b}dt$$

Then, by substituting Equation (18) into Equation (14), $\phi_{\text{2}}^{n}$ can be expressed as follows:

(A12) $$\begin{array}{c} \phi_{\text{2}}^{n}=-C_{i_{b0}}^{n}\left(t\right)\int_{0}^{t}C_{b}^{i_{b0}}\left(t\right)\varepsilon^{b}dt\\ =-C_{i}^{n}\left(t\right)C_{i_{b0}}^{i}\int_{0}^{t}C_{b}^{i_{b0}}\left(t\right)\varepsilon^{b}dt\\ =-C_{i}^{n}\left(t\right)\int_{0}^{t_{k}}C_{i_{b0}}^{i}C_{n}^{i_{b0}}\left(t\right)C_{b}^{n}\varepsilon^{b}dt\\ =-C_{i}^{n}\left(t\right)\int_{0}^{t_{k}}C_{n}^{i}\left(t\right)\varepsilon^{n}dt \end{array}$$

where

(A13) $$C_{i}^{n}\left(t\right)\text{=}C_{e}^{n}C_{i}^{e}(t)\text{=}\left[\begin{array}{ccc} \cos\left(\omega_{ie}t\right) & \sin\left(\omega_{ie}t\right) & 0\\ -\sin L\sin\left(\omega_{ie}t\right) & \sin L\cos\left(\omega_{ie}t\right) & \cos L\\ \cos L\sin\left(\omega_{ie}t\right) & \text{-}\cos L\cos\left(\omega_{ie}t\right) & \sin L \end{array}\right]$$

(A14) $$C_{n}^{i}\left(t\right)={\left(C_{i}^{n}\left(t\right)\right)}^{T}=\left[\begin{array}{ccc} \cos\left(\omega_{ie}t\right) & -\sin L\sin\left(\omega_{ie}t\right) & \cos L\sin\left(\omega_{ie}t\right)\\ \sin\left(\omega_{ie}t\right) & \sin L\cos\left(\omega_{ie}t\right) & -\cos L\cos\left(\omega_{ie}t\right)\\ 0 & \cos L & \sin L \end{array}\right]$$

(A15) $$\varepsilon^{n}={\left[\begin{array}{ccc} \varepsilon_{e} & \varepsilon_{n} & \varepsilon_{u} \end{array}\right]}^{T}$$

Then, substituting Equations (A13)–(A15) into Equation (A12), we obtain:

(A16) $$\phi_{\text{2}}^{n}\text{=}\left[\begin{array}{c} -\frac{\sin L\cdot \varepsilon_{n}}{\omega_{ie}}+\frac{\cos L\cdot \varepsilon_{u}}{\omega_{ie}}\\ \frac{\sin L\cdot \varepsilon_{e}}{\omega_{ie}}-\cos^{2}L\cdot t\cdot \varepsilon_{n}-\cos L\cdot \sin L\cdot t\cdot \varepsilon_{u}\\ -\frac{\cos L\cdot \varepsilon_{e}}{\omega_{ie}}-\cos L\cdot \sin L\cdot t\cdot \varepsilon_{n}-\sin^{2}L\cdot t\cdot \varepsilon_{u} \end{array}\right]$$

Next, let us analyze the error of $C_{i_{b0}}^{i}$. In practice, accelerometer measurements contain interference error. Therefore, the $g^{i_{b0}}$ used in Equation (5) is relatively difficult to get. For purposes of analysis, consider the static base condition; let ${\hat{f}}^{b}=f^{b}+\nabla^{b}$ , where $\nabla^{b}$ is the accelerometer bias. If neglecting the second-order small items, we have

(A17) $${\hat{f}}^{i_{b0}}={\hat{C}}_{b}^{i_{b0}}{\hat{f}}^{b}=\left[I-(\phi^{i_{b0}}\times )\right]C_{b}^{i_{b0}}\left(f^{b}+\nabla^{b}\right)\approx f^{i_{b0}}-(\phi^{i_{b0}}\times )f^{i_{b0}}+\nabla^{i_{b0}}$$

Then, substitute Equation (A17) into Equation (5) and define

(A18) $${\left({\text{G}}^{i}\right)}^{-1}={\left[\begin{array}{c} {\left[g^{i}(t_{1})\right]}^{T}\\ {\left[g^{i}(t_{2})\right]}^{T}\\ {\left[g^{i}(t_{1})\times g^{i}(t_{2})\right]}^{T} \end{array}\right]}^{-1}$$

(A19) $$F^{i_{b0}}=[\begin{array}{c} {\left[f^{i_{b0}}(t_{1})\right]}^{T}\\ {\left[f^{i_{b0}}(t_{2})\right]}^{T}\\ {\left[f^{i_{b0}}(t_{1})\times f^{i_{b0}}(t_{2})\right]}^{T} \end{array}]$$

(A20) $${\hat{F}}^{i_{b0}}=[\begin{array}{c} {\left[{\hat{f}}^{i_{b0}}(t_{1})\right]}^{T}\\ {\left[{\hat{f}}^{i_{b0}}(t_{2})\right]}^{T}\\ {\left[{\hat{f}}^{i_{b0}}(t_{1})\times {\hat{f}}^{i_{b0}}(t_{2})\right]}^{T} \end{array}]$$

(A21) $${\hat{F}}^{i_{b0}}=F^{i_{b0}}+\delta F^{i_{b0}}$$

Substitute Equation (A17) into Equation (A20) and ignore second-order small item, and we get

(A22) $$\delta F^{i_{b0}}\text{=}{\hat{F}}^{i_{b0}}\text{-}F^{i_{b0}}\approx [\begin{array}{c} {\left[\nabla^{i_{b0}}-\phi^{i_{b0}}(t_{1})\times f^{i_{b0}}(t_{1})\right]}^{T}\\ {\left[\nabla^{i_{b0}}-\phi^{i_{b0}}(t_{2})\times f^{i_{b0}}(t_{2})\right]}^{T}\\ {\{\left[\nabla^{i_{b0}}-\phi^{i_{b0}}(t_{1})\times f^{i_{b0}}(t_{1})\right]\times f^{i_{b0}}(t_{2})+f^{i_{b0}}(t_{1})\times \left[\nabla^{i_{b0}}-\phi^{i_{b0}}(t_{2})\times f^{i_{b0}}(t_{2})\right]\}}^{T} \end{array}]$$

According to Equation (6), we know that ${\left(G^{i}\right)}^{-1}$ will not introduce any error during the calculation process. Therefore,

(A23) $${\hat{C}}_{i_{b0}}^{i}={\left(G^{i}\right)}^{-1}{\hat{F}}^{i_{b0}}={\left(G^{i}\right)}^{-1}F^{i_{b0}}+{\left(G^{i}\right)}^{-1}\delta F^{i_{b0}}=(I+{\left(G^{i}\right)}^{-1}\delta F^{i_{b0}}C_{i}^{i_{b0}})C_{i_{b0}}^{i}$$

According to reference [1], we have

(A24) $${\hat{C}}_{i_{b0}}^{i}={\hat{C}}_{i_{b0}}^{i}{\left[{\left({\hat{C}}_{i_{b0}}^{i}\right)}^{T}{\hat{C}}_{i_{b0}}^{i}\right]}^{-\frac{1}{2}}\approx \left[I-\frac{1}{2}\left({\left({\left(G^{i}\right)}^{-1}\delta F^{i_{b0}}C_{i}^{i_{b0}}\right)}^{T}-\left(G^{i}\right)-1\delta F^{i_{b0}}C_{i}^{i_{b0}}\right)\right]C_{i_{b0}}^{i}$$

Compare Equations (A4) and (A24), we know that

(A25) $$\left(\phi^{i}\times \right)\text{=}\frac{\text{1}}{\text{2}}\left({\left({\left(G^{i}\right)}^{-1}\delta F^{i_{b0}}C_{i}^{i_{b0}}\right)}^{T}-{\left(G^{i}\right)}^{-1}\delta F^{i_{b0}}C_{i}^{i_{b0}}\right)$$

Since $C_{i}^{i_{b0}}$ is an orthonormal matrix, when Equation (A17) is substituted into Equation (5) we get

(A26) $$\delta F^{i_{b0}}C_{i}^{i_{b0}}=[\begin{array}{c} {[\nabla^{i}-\phi^{i}(t_{1})\times f^{i}(t_{1})}^{T}]\\ {\left[\nabla^{i}-\phi^{i}(t_{2})\times f^{i}(t_{2})\right]}^{T}\\ {\{\left[\nabla^{i}-\phi^{i}(t_{1})\times f^{i}(t_{1})\right]\times f^{i}(t_{2})+f^{i}(t_{1})\times \left[\nabla^{i}-\phi^{i}(t_{2})\times f^{i}(t_{2})\right]\}}^{T} \end{array}]=\delta F_{A}^{i}+\delta F_{G}^{i}$$

Through an analysis of Equation (A26), we find that $\delta F^{i_{b0}}C_{i}^{i_{b0}}$ consists of two parts; the first part is caused by accelerometer error, and the second part is caused by gyro error, where

(A27) $$\delta F_{A}^{i}=\left[\begin{array}{c} {\left(\nabla^{i}\right)}^{T}\\ {\left(\nabla^{i}\right)}^{T}\\ {\left[\nabla^{i}\times f^{i}(t_{2})+f^{i}(t_{1})\times \nabla^{i}\right]}^{T} \end{array}\right]$$

(A28) $$\delta F_{G}^{i}=-[\begin{array}{c} {\left[\phi^{i}(t_{1})\times f^{i}(t_{1})\right]}^{T}\\ {\left[\phi^{i}(t_{2})\times f^{i}(t_{2})\right]}^{T}\\ {\{\left[\phi^{i}(t_{1})\times f^{i}(t_{1})\right]\times f^{i}(t_{2})+f^{i}(t_{1})\times \left[)\times \left[\phi^{i}(t_{2})\times f^{i}(t_{2})\right]\right\}}^{T} \end{array}]$$

According to the principle of coordinate transformation we have

(A29) $$\nabla^{i}\text{=}C_{n}^{i}\nabla^{n}$$

(A30) $$\left[\phi^{i}\times f^{i}\right]=-\left(\int_{0}^{t}\left[\varepsilon^{i}\times \right]dt\right)C_{n}^{i}g^{n}$$

(A31) $$\varepsilon^{i}=C_{n}^{i}\varepsilon^{n}$$

Under the static conditions, $f^{i}=g^{i}$, if we introduce Equations (34)–(38) into Equation (32), and assume sin(ωt)≈0, cos(ωt)≈1, we finally obtain the errors $\phi_{g}^{i}$ and $\phi_{a}^{i}$ caused by gyros and accelerometers, respectively. Then, project them into the navigation frame, and we have

(A32) $$\phi_{\text{3}a}^{n}={\left[\begin{array}{ccc} -\frac{\nabla_{n}}{g} & \frac{\sin L\cdot \omega_{ie}t\nabla_{n}}{g}+\frac{\nabla_{e}}{g} & -\frac{\cos L\cdot \omega_{ie}t\nabla_{n}}{g}+\frac{\nabla_{e}}{g}\tan L \end{array}\right]}^{T}$$

(A33) $$\phi_{\text{3}g}^{n}={\left[\begin{array}{ccc} \frac{\sin L\cdot \varepsilon_{n}}{\omega_{ie}}-\frac{\cos L\cdot \varepsilon_{u}}{\omega_{ie}} & -\frac{\varepsilon_{e}\sin L}{\omega_{ie}}+\frac{\varepsilon_{n}t}{2} & -\frac{\sin L^{2}\varepsilon_{e}}{\cos L\omega_{ie}}+\varepsilon_{u}t \end{array}\right]}^{T}$$

According to Equations (A16), (A32) and (A33), we can get the following result: the misalignment angle caused by gyro error is

(A34) $$\phi_{g}^{n}=\phi_{\text{2}}^{n}\text{+}\phi_{\text{3}g}^{n}\text{=}{\left[\begin{array}{ccc} \text{0} & (\frac{1}{2}-\cos^{2}L)\varepsilon_{n}t+\sin L\cos L\varepsilon_{u}t & \cos^{2}L\cdot \varepsilon_{u}t-\sin L\cos L\varepsilon_{n}t-\frac{\varepsilon_{e}}{\cos L\cdot \omega_{ie}} \end{array}\right]}^{T}$$

The misalignment angle caused by accelerometers’ error is expressed as Equation (A32).

For further analysis, assume that t=360s, $\nabla_{e}=\nabla_{n}=\nabla_{u}=1\times 10^{-4}g$, $\varepsilon_{e}{=\varepsilon }_{n}{=\varepsilon }_{u}=0.01^{\circ }\text{/}h$. It is also known that $\omega_{ie}\approx 7.3\times 10^{-5}rad/s$ and $L\in [0,\frac{\pi }{2}]$. Therefore, it can be calculated approximately and we find that the error terms consisting of t are very small and can be ignored. Therefore,

(A35) $$\phi^{n}=\phi_{\text{3}a}^{n}+\phi_{g}^{n}\approx \left[\begin{array}{c} -\frac{\nabla_{n}}{g}\\ \frac{\nabla_{e}}{g}\\ -\frac{\varepsilon_{e}}{\cos L\cdot \omega_{ie}}\text{+}\frac{\nabla_{e}}{g}\tan L \end{array}\right]$$

Through analysis, we know that the pitch angle error and the roll angle error depend on the accelerometer bias. The yaw angle error is determined by equivalent east gyro drift, equivalent east accelerometer bias and latitude.

### B. Gaussian Approximation Filters

Consider the following discrete-time nonlinear state-space model:

(B1) $$x_{k+1}=f(x_{k})+w_{k}$$

(B2) $$y_{k}=h(x_{k})+v_{k}$$

where, k∈{1,2,…} is discrete time, $x_{k}$ and $y_{k}$ are the state vector and the measurement vector at time k, respectively; $w_{k}$ and $v_{k}$ are the noise vectors from two independent zero-mean Gaussian processes with their covariance matrices $Q_{k}$ and $R_{k}$, respectively .

Under the assumption of Gaussian distributions, the general form of Gaussian approximation filtering algorithm can be summarized as follows:

(B3) $${\hat{x}}_{k+1}={\hat{x}}_{k+1/k}+K_{k+1}(y_{k+1}-{\hat{y}}_{k+1})$$

(B4) $$K_{k+1}=P_{k+1/k}^{xy}{\left(P_{k+1}^{y}\right)}^{-1}$$

(B5) $$P_{k+1}=P_{k+1/k}-K_{k+1}P_{k+1}^{y}K_{k+1}^{T}$$

where, ${\hat{x}}_{k+1}$ is the estimation of the state; ${\hat{y}}_{k+1}$ is the estimation of the measurement with the covariance matrix $P_{k+1}^{y}$; ${\hat{x}}_{k+1/k}$ is the state prediction; $K_{k+1}$ is the gain matrix; $P_{k+1/k}^{xy}$ is the cross covariance matrix; $N(x_{k};{\hat{x}}_{k},P_{k})$ denotes the multivariate normal distribution with mean ${\hat{x}}_{k}$ and covariance $P_{k}$

(B6) $$P_{k+1}=P_{k+1/k}-K_{k+1}P_{k+1}^{y}K_{k+1}^{T}$$

(B7) $$P_{k+1/k}=\int_{R^{n}}f(x_{k})f^{T}(x_{k})N(x_{k};{\hat{x}}_{k},P_{k})dx_{k}-{\hat{x}}_{k+1/k}{\hat{x}}_{k+1/k}^{T}+Q_{k}$$

(B8) $${\hat{y}}_{k+1/k}=\int_{R^{n}}h(x_{k+1})N(x_{k+1};{\hat{x}}_{k+1/k},P_{k+1/k})dx_{k+1}$$

(B9) $$P_{k+1}^{y}=\int_{R^{n}}h(x_{k+1})h^{T}(x_{k+1})N(x_{k+1};{\hat{x}}_{k+1/k},P_{k+1/k})dx_{k+1}-{\hat{y}}_{k+1/k}{\hat{y}}_{k+1/k}^{T}+R_{k+1}$$

(B10) $$P_{k+1/k}^{xy}=\int_{R^{n}}x_{k+1}h^{T}(x_{k+1})N(x_{k+1};{\hat{x}}_{k+1/k},P_{k+1/k})dx_{k+1}\;-{\hat{x}}_{k+1/k}{\hat{y}}_{k+1/k}^{T}$$

The integrals in can be approximated by the GHQ, UT, or cubature rule [20,22,23]. The generic quadrature-based Gaussian approximation filter is given as [22,23].

Prediction:

(B11) $${\hat{x}}_{k+1/k}=\sum_{i=1}^{N_{p}}w_{i}f\left(\varepsilon_{i}\right)$$

(B12) $$P_{k+1/k}=\sum_{i=1}^{N_{p}}w_{i}\left[f\left(\varepsilon_{i}\right)-{\hat{x}}_{k+1/k}\right]{\left[f\left(\varepsilon_{i}\right)-{\hat{x}}_{k+1/k}\right]}^{T}+Q_{k}$$

where, $N_{p}$ is the total number of points $\varepsilon_{i}$ is the transformed point obtained from the covariance decomposition such as Cholesky decomposition; that is

(B13) $$P_{k}=SS^{T}$$

(B14) $$\varepsilon_{i}=S\gamma_{i}+{\hat{x}}_{k}$$

$\gamma_{i}$ is the point corresponding to N(x;0,I); and $w_{i}$ is the associated weight.

Update:

(B15) $${\hat{x}}_{k+1}={\hat{x}}_{k+1/k}+K_{k+1}(y_{k+1}-{\hat{y}}_{k+1})$$

(B16) $$P_{k+1}=P_{k+1|k}-K_{k}P_{k+1/k}^{xy}$$

where

(B17) $${\hat{y}}_{k+1/k}=\sum_{i=1}^{N_{p}}w_{i}h\left({\tilde{\varepsilon }}_{i}\right)$$

(B18) $$P_{k+1/k}^{xy}=\sum_{i=1}^{N_{p}}w_{i}\left({\tilde{\varepsilon }}_{i}-{\hat{x}}_{k+1/k}\right){\left[h({\tilde{\varepsilon }}_{i})-{\hat{y}}_{k+1/k}\right]}^{T}$$

(B19) $$P_{k+1}^{y}=\sum_{i=1}^{N_{p}}w_{i}\left[h({\tilde{\varepsilon }}_{i})-{\hat{y}}_{k+1/k}\right]{\left[h({\tilde{\varepsilon }}_{i})-{\hat{y}}_{k+1/k}\right]}^{T}+R_{k+1}$$

where ${\tilde{\varepsilon }}_{i}$ is the transformed point obtained from the predicted covariance decomposition; that is

(B20) $$P_{k+1|k}=\tilde{S}{\tilde{S}}^{T}$$

(B21) $${\tilde{\varepsilon }}_{i}=\tilde{S}\gamma_{i}+{\hat{x}}_{k+1/k}$$

### C. Gauss-Hermite Quadrature Point Selection

For the univariate standard Gaussian distribution with m quadrature points $\left(\gamma_{i},w_{i}\right)$ can be calculated as follows [23,25].

If m=1, then $\left(\gamma_{i},w_{i})=(0,1\right)$.

If m>1, firstly, a symmetric tridiagonal matrix J needs to be constructed, whose diagonal elements are zero and $J_{i,i+1}$ = $\sqrt{\frac{i}{2}}$ 1≤i≤m−1). Then the quadrature point $\left(\gamma_{i},w_{i})=(\sqrt{2}\xi_{i},{{\left(v_{i}\right)}_{1}}^{2}\right)$, where $\xi_{i}$ is the i-th eigenvalue of J, ${\left(v_{i}\right)}_{1}$ is the first element of the i-th normalized eigenvector of J.

For example, m=3, we have $(\gamma_{i},w_{i})=\left\{\left(-\sqrt{3},\frac{1}{6}),(0,\frac{2}{3}),(\sqrt{3},\frac{1}{6}\right)\right\}$.

The multivariate GHQ rule extends the univariate m-point set to the n-dimensional point set by the tensor product rule. However, the total number of points is $N_{p}=m^{n}$, which increases exponentially with dimension n. That will result in the so-called” curse of dimensionality”.

### D. RMS Error

RMS error means the square root of the mean/average of the square of all of the error. It is commonly used and makes an excellent general purpose error metric. The calculation formula is as follows:

(D1) $$RMSE=\sqrt{\frac{1}{N}\sum_{i=1}^{N}{\left(E_{i}-\bar{E}\right)}^{2}}$$

where N is the number of experiments, in this paper N=100, $E_{i}$ is the *i*-th experimental result, and $\bar{E}$ denotes arithmetic mean value of all the experimental results .

## References

[1] Britting K.R. (1971). Inertial Navigation Systems Analysis.

[2] Titterton D., Weston J.L. (2004). Strapdown Inertial Navigation Technology.

[3] Qin Y., Yan G., Gu D., Zheng J. (2005). Clever way of sins coarse alignment despite rocking ship. Xibei Gongye Daxue Xuebao/J. Northwest. Polytech. Univ..

[4] Sun F., Lan H.Y., Yu C.Y., El-Sheimy N., Zhou G.T., Cao T., Liu H. (2013). A robust self-alignment method for ship’s strapdown INS under mooring conditions. Sensors.

[5] Gu D.Q., El-Sheimy N., Hassan T., Syed Z. Coarse alignment for marine sins using gravity in the inertial frame as a reference. Proceedings of the 2008 IEEE/ION Position, Location and Navigation Symposium.

[6] Silson P.M.G. (2011). Coarse alignment of a ship’s strapdown inertial attitude reference system using velocity loci. IEEE Trans. Instrum. Meas..

[7] Sun F., Sun W. (2010). Mooring alignment for marine SINS using the digital filter. Measurement.

[8] Gao W., Ben Y.Y., Zhang X., Li Q., Yu F. (2011). Rapid fine strapdown ins alignment method under marine mooring condition. IEEE Trans. Aerosp. Electron. Syst..

[9] Gao W., Che Y., Zhang X., Feng J., Zhang B. A fast alignment algorithm based on inertial frame for marine SINS. Proceedings of the 2012 9th IEEE International Conference on Mechatronics and Automation (ICMA 2012).

[10] Hong H.S., Lee J.G., Park C.G. (2002). In-flight alignment of SDINS under large initial heading error. Trans. Soc. Instrum. Control Eng..

[11] Wei C., Zhang H. (2001). Sins in-flight alignment using quaternion error models. Chinese J. Aeronaut..

[12] Yu M.-J., Park H.-W., Jeon C.-B. Equivalent nonlinear error models of strapdown inertial navigation system. Proceedings of the 1997 AIAA Guidance, Navigation, and Control Conference.

[13] Dmitriyev S.P., Stepanov O.A., Shepel S.V. (1997). Nonlinear filtering methods application in ins alignment. IEEE Trans. Aerosp. Electron. Syst..

[14] Wang B., Xiao X., Xia Y.Q., Fu M.Y. (2013). Unscented particle filtering for estimation of shipboard deformation based on inertial measurement units. Sensors.

[15] Guo L., Cao S.Y., Qi C.T., Gao X.Y. (2012). Initial alignment for nonlinear inertial navigation systems with multiple disturbances based on enhanced anti-disturbance filtering. Int. J. Control..

[16] Kubo Y., Fujioka S., Nishiyama M., Sugimoto S. (2006). Nonlinear filtering methods for the INS/GPS in-motion alignment and navigation. Int J. Innov. Comput. Inf. Control.

[17] Gao W., Che Y., Yu F., Liu Y. A fast inertial frame alignment algorithm based on horizontal alignment information for marine SINS. Proceedings of the 2014 IEEE/ION Position, Location and Navigation Symposium-PLANS 2014.

[18] Anderson B.D., Moore J.B. (1979). Optimal Filtering.

[19] Smith G.L., Schmidt S.F., McGee L.A. (1962). Application of Statistical Filter Theory to the Optimal Estimation of Position and Velocity on Board a Circumlunar Vehicle.

[20] Julier S.J., Uhlmann J.K. (2004). Unscented filtering and nonlinear estimation. Proc. IEEE.

[21] Arasaratnam I., Haykin S., Hurd T.R. (2010). Cubature kalman filtering for continuous-discrete systems: Theory and simulations. IEEE Trans. Signal. Process..

[22] Arasaratnam I., Haykin S. (2009). Cubature kalman filters. IEEE Trans. Autom. Control..

[23] Ito K., Xiong K. (2000). Gaussian filters for nonlinear filtering problems. IEEE Trans. Autom. Control..

[24] Gao W., Zhang Y., Wang J.G. (2014). A strapdown interial navigation system/beidou/doppler velocity log integrated navigation algorithm based on a cubature kalman filter. Sensors.

[25] Arasaratnam I., Haykin S., Elliott R.J. (2007). Discrete-time nonlinear filtering algorithms using gauss-hermite quadrature. Proc. IEEE.

[26] Jia B., Xin M., Cheng Y. Sparse gauss-hermite quadrature filter for spacecraft attitude estimation. Proceedings of the 2010 American Control Conference.

[27] Jia B., Xin M., Cheng Y. (2013). High-degree cubature kalman filter. Automatica.

